# Effect of antihypertensive therapy on cognitive functions of patients with hypertension

**DOI:** 10.4103/0972-2327.70880

**Published:** 2010

**Authors:** Ashok Jaiswal, V. Bhavsar, Jaykaran, N. D. Kantharia

**Affiliations:** Marketing, Zydus Cedilla, Ahmedabad India; 1 Department of Pharmacology, New Civil Hospital, Government Medical College, Majura Gate, Surat-395 001, India

**Keywords:** Antihypertensive therapy, cognitive functions, hypertension

## Abstract

**Objectives:**

Hypertension is known to be associated with cognitive decline. Many studies revealed that control of hypertension with antihypertensive therapy controls the cognitive decline associated with hypertension. While there are reports that suggest that antihypertensive drugs do not provide protection from cognitive decline, the present study is designed to evaluate the cognitive status of patients recently diagnosed as hypertensive and effect of 3 month long antihypertensive therapy on cognitive functions.

**Materials and Methods:**

A predesigned pretested questionnaire was used to collect the information. The PGI memory scale (PGIMS) was employed to assess memory function of patients. Baseline memory functions were evaluated before starting the treatment with antihypertensive and compared with the cognitive function scores of healthy volunteers. After the 3 months of treatment, cognitive functions were evaluated again by the same scale. The unpaired t-test was used to compare the cognitive functions between case and control and the paired *t*-test was used to compare pre- and post-treatment score.

**Results:**

This study revealed that mean scores of six subtests of cognitive functions were less in cases as compared to subjects in comparison group. After 3 months of antihypertensive therapy, scores of five sub-tests were found to be increased. Among these five subtests, four were those which were found declined at the baseline.

**Conclusion:**

This suggests that antihypertensive therapy given for 3 months improved the score of those cognitive function tests in which hypertensive patients perform poorly during recruitment and there was no deterioration of any test after 3 months of antihypertensive therapy.

## Introduction

Hypertension is the most common cardiovascular disease. The prevalence of hypertension increases with age. Elevated arterial pressure is associated with progressive pathological changes which leads to various cardiovascular and central nervous system-related complications such as stroke,[1] vascular dementia,[2] and probably Alzheimer’s disease.[3] Uncontrolled hypertension may lead to cognitive decline.[4] Hypertension brings certain pathophysiological changes in brain such as vascular remodeling, impaired cerebral auto regulation, small lacunar infarct, white matter lesion, microbleed and amyloid angiopathy, etc. which may result in deterioration of the cognitive functions.[5]

Many studies revealed that control of hypertension with antihypertensive therapy controls the cognitive decline associated with hypertension.[6] Nevertheless, there are reports which suggest that antihypertensive drugs do not provide protection from cognitive decline.[7]

The present study is designed to evaluate cognitive status of patients recently diagnosed as hypertensive and compare it with suitable subjects in comparison group and to see the effect of antihypertensive treatment on cognitive functions of patients of hypertension.

### Patient selection

#### Selection of cases and comparison group

Cases: Patients who were recently diagnosed as hypertensive and were prescribed antihypertensive therapy by their treating physician.

Inclusion criteria for the cases were patients recently diagnosed as hypertensive (SBP ≥ 140 mmHg and/or DBP ≥ 90 mmHg), Age: between 20 and 60 years, Sex: both males and females and: ability to understand, read, write, and communicate in Gujarati with primary knowledge of English.

Exclusion criteria for the cases were patients with prehypertension (SBP 120-139 mmHg and DBP 80-89), age less than 20 and more than 60, patients known to have psychological and behavioral disorders or any other CNS disorder that could interfere with the memory and psychomotor functions and patients on any other medications (e.g. sedatives, antipsychotics, antidepressants, antihistaminic) that are known to affect memory and psychomotor functions.

The comparison group consisted of persons who satisfied the following criteria:

Inclusion criteria for comparison group were patietns with blood pressure within normal and pre-hypertension range, sex: both males and females, age: between 20 and 60 years and ability to understand, read, write, and communicate in Gujarati with primary knowledge of English.

Exclusion criteria for comparison group were persons diagnosed as hypertensive (SBP≥ 140 mmHg, DBP≥ 90 mmHg), known to have psychological and behavioral disorders or any other CNS disorder that could interfere with the memory and psychomotor functions and patients on any other medications (e.g. sedatives, antipsychotics, antidepressants, antihistaminic) that are known to affect memory and psychomotor functions.

## Materials and Methods

This study was designed to evaluate the effect of antihypertensive therapy for a period of 3 months on cognitive functions (memory and psychomotor) in patients having hypertension.

### Study tool

A predesigned pretested questionnaire was used to collect the information. The PGI memory scale (PGIMS)[8,9] was employed to assess memory function of patients.

### Methodology

Between May and July 2005, the investigator visited the OPD of private physician, each day in the morning from 9:00 A.M to 1:00 P.M for screening the patients. The study was started after the permission of institutional ethics committee. Those who met with the inclusion and exclusion criteria were enrolled in the study group. The aim and procedure of the study and the tests were explained to the subjects. Writt en informed consent was obtained from the patient. The baseline information was collected on the day zero i.e. before starting of the drug treatment. The patients were then evaluated with the help of PGI memory scale after 3 months. Subjects in the comparison group were recruited from the class III and class IV employee of Govt. medical college, Surat. They were evaluated only once i.e. on the day of recruitment. Before applying tests for cognitive functions, the systolic and diastolic blood pressure was recorded by the auscultatory method with the help of sphygmomanometer as average of two readings in the sitt ing position corrected to 2 mmHg in study group at both the occasions, i.e. during recruitment and then subsequent assessment after 3 months. In the control group blood pressure was measured at the time of recruitment.

All the cognitive functions tests were conducted in Gujarati language. They were conducted in particular sequence and this sequence was maintained for every subject.

### Tests

#### Tests for memory

The PGI memory scale (PGIMS)[8,9] was employed to assess memory function of patients. PGI memory scale consists of ten sub-tests. These tests measure different aspects of memory and employ different methods of recall. These tests are –

**Remote memory:** It comprises six simple questions relating to the past events of personal life.

**Recent memory:** It consists of five questions that assess the patient’s ability to recall information and events in the recent past.

**Immediate recall:** This test includes sequential reproduction of the sentences in verbatim.

**Mental balance:** This test gives an idea of balance over ones mental functioning.

**Attention and concentration:** This function was evaluated by the test of digit span forward and backward repetition.

**Word list memory test:** The patients were instructed to recall the common objects after 1 minute.

**Paired associate test:** Patients ability to associate with similar and dissimilar words were checked.

**Rays figure test:** Subjects were instructed to draw a geometrical figure which was already shown to them for 30 seconds.

**Recognition:** Patient were instructed to recognize the previously shown common pictures from a large set of pictures.

**Six Letter Cancellation Test (SLCT):**[10] It is a psychomotor function test in which perceptual processing of sensory information can be readily assessed. Patients were asked to cancel as many as many target alphabets as possible within 90 seconds.

**Line test:** This test comprises judgment of length of a line and drawing the line of same length.

**Delayed recall test:** Subjects were asked to recall the three sentences of earlier immediate recall test. So it is the extension of immediate recall test.

### Statistical analysis

Data obtained in the various tests were analyzed using SPSS version 12. Analysis of distribution of data was done using the Komolgorov--Smirnov test and Shapiro--Wills test. The unpaired Student *t*-test was used to compare blood pressure and cognitive function between case and control. The paired *t*-test was used to compare blood pressure and cognitive functions at baseline and after 3 months.

## Results

To see the effect of hypertension on cognitive functions, the cognitive functions were compared between hypertensive patients (study group) and healthy volunteers (comparison group). A total of 50 subjects were enrolled in each group [Table 1].

**Table 1:** Baseline characteristics of case and comparison group

	Cases *n*=50	Comparison group *n*=50	*P* value
Age (years)	51.02 ± 7.7	46.0 ± 7.1	> 0.05
SBP (mmHg)	149.1 ± 6.73	123.05 ± 6.2	< 0.001
DBP (mmHg)	98.71 ± 3.7	78 ± 4.4	< 0.001
Education (in years)			> 0.05
5-10 years	2(4)	1 (2)	
11-12 years	9 (18)	7 (14)	
University graduation	37 (74)	41 (82)	
University Post graduation	2 (4)	1 (2)	

(All data are expressed in mean±SD, value in parenthesis are percentage)

The unpaired *t*-test was used to see the difference between age, SBP and DBP between two groups. Differences between educational qualifications were analyzed by Fisher’s exact test.

The mean systolic blood pressure and mean diastolic blood pressures were statistically different between the study and control population. It was observed that all the cases were prescribed atenolol in the dose of 25-50 mg/day. Some of the patients were prescribed other antihypertensives like amlodipine (*n*=5), chlorthalidone (*n*=7), and antiplatelets like low dose aspirin (*n*=14) or clopidogrel (*n*=6).

There is a significant difference in immediate recall test, mental balance test, forward digit span test, word list memory test, recognition test and six-letter cancellation test between the study and comparison group [Table 2].

**Table 2:** Comparative test score showing cognitive functions between cases and comparison group

Test	Cases group score	Comparison group score
Remote memory	5.82 ± 0.44	5.81 ± 0.44
Recent memory	5.00 ± 0.00	4.95 ± 0.20
Immediate recall	8.80 ± 1.28	9.77 ± 1.50***
Mental balance	6.13 ± 0.96	6.63 ± 1.12*
Forward digit span	4.77 ± 0.79	5.15 ± 0.96*
Backward digit span	3.28 ± 0.66	3.43 ± 0.69
Word list memory	4.88 ± 0.95	5.63 ± 0.83***
Paired associate test	3.13 ± 0.99	3.47 ± 1.32
Ray’s figure test	5.82 ± 2.08	6.31 ± 1.73
Recognition	10.73 ± 1.07	11.34 ± 0.88**
Six-letter cancellation test	14.11 ± 3.9	17.31 ± 3.48***
Line test	8.48 ± 0.75	8.34 ± 0.91
Delayed recall test	3.48 ± 1.16	3.84 ± 1.41

(All data are expressed in mean±SD);

* *P* value < 0.05;

** *P* value < 0.01;

*** *P* value < 0.001; Unpaired *t*-test

### Effect of antihypertensive therapy on cognitive functions of hypertensive patients

Baseline memory tests and blood pressure of cases was measured twice [Table 3]. At the beginning of treatment, (at day one), and at the end of 3 months. Adherence was assured by checking the empty blister packet and checking the unused medicines. Five subjects were lost to follow up during the study. It was seen that there was a significant decrease in both systolic and diastolic blood pressure after 3 months of follow up.

**Table 3:** Cognitive function test scores and blood pressure of cases at the start of therapy and after the follow-up of 3 months

Test	Study group initial score (*n*=50)	Study group score after 3 months (*n*=45)
Remote memory	5.82 ± 0.44	5.75 ± 0.48
Recent memory	5.00 ± 0.00	4.97 ± 0.14
Immediate recall	8.80 ± 1.28	9.42 ± 1.28***
Mental balance	6.13 ± 0.96	6.28 ± 0.96
Forward digit span	4.77 ± 0.79	4.88 ± 0.48
Backward digit span	3.28 ± 0.66	3.31 ± 0.55
Word list memory	4.88 ± 0.95	5.17 ± 0.77*
Paired associate test	3.13 ± 0.99	3.13 ± 0.97
Ray’s figure test	5.82 ± 2.08	6.13 ± 1.7
Recognition	10.73 ± 1.07	11.42 ± 0.72***
Six-letter cancellation test	14.11 ± 3.9	16.77 ± 3.57***
Line test	8.48 ± 0.75	8.44 ± 0.69
Delayed recall test	3.48 ± 1.16	4.06 ± 1.13***
SBP (mmHg)	149.1 ± 6.73	132.97 ± 7.77***
DBP (mmHg)	98.71 ± 3.7	84.62 ± 4.62***

(All data are expressed in mean±SD);

* *P* value < 0.05;

**P value < 0.01;

*** *P* value < 0.01; Paired *t*-test

After 3 months of therapy of antihypertensives, there was significant improvement in immediate recall test, recognition test, six-letter cancellation test, word list memory test, and delayed recall test.

During comparison of subjects in cases and comparison group, it was found that there was decline in cognitive functions as indicated by immediate recall test, mental balance test, forward digit span test, word list memory test, recognition test, and sixletter cancellation test. Out of these tests, immediate recall test, word list memory test, recognition, and six-letter cancellation test showed improvement after 3 months of antihypertensive therapy.

Some tests like remote memory test, recent memory test, backward digit span test, paired associate test, Ray’s figure test, line test, and delayed recall test showed no decline. Out of these, only delayed recall test showed improvement after 3 months of antihypertensive therapy.

## Discussion

In this study, the baseline cognitive functions of patients who were recently diagnosed with hypertension were found to be declined as compared to the comparison group.

It was seen that mean scores of six subtests of cognitive functions were less as compared to subjects in the comparison group. This decline may be because of pathological effect of hypertension. The relationship between diagnosis of hypertension and cognitive functions were explored in other studies.[11] There are other studies which shows that poor cognitive functions are because of pathological changes of hypertension.[4,5,12]

After 3 months of antihypertensive therapy, scores of five subtests were found to be increased. Among these five sub-tests, four were those which found declined at the baseline. This suggests that antihypertensive therapy for 3 months improve the score of those cognitive function tests in which hypertensive patients perform poorly during recruitment in comparison to the subjects of the control group. There was no deterioration of any test after 3 months of antihypertensive therapy.

It is observed that high blood pressure is associated with the various pathological changes in brain and one of the important changes is alteration in cerebral blood flow.[13] In a study done by Lipsitz et al. (2005) it was found that 6 month antihypertensive therapy is associated with increase in the cerebral blood flow.[14] Similar findings were also observed in other studies.[15] Short-term administration of some antihypertensive drugs such as beta blockers and ACE inhibitors are also associated with improvement in cerebral blood flow in patients of hypertension.[16] The improvement in cognition and memory in patients of this study may be because of improvement in cerebral blood flow though there is a need of study with larger sample size with concomitant measure of cerebral blood flow to validate this finding.

In the present study, it was observed that that blood pressure was controlled within 3 months of antihypertensive treatment and no deterioration in cognitive functions was observed. The finding of this study does not correlate with study of Bellew et al. (2004). They observed that patients younger than 65 year with hypertension were more likely to have increased cognitive decline as compared to nonhypertensive control. But the treatment with antihypertensive did not appear to provide protection from cognitive decline.[7]

All the cases received atenolol as one of the drug. Atenolol is a beta-1 selective antagonist. It is very hydrophilic and appear to penetrate the brain only to limited extent.[17] Despite hydrophilic nature CNS effects of atenolol have been documented.[18] We found no deleterious effects of beta blockers on cognition and memory as reported by some studies.[18]

In present study, other antihypertensives (amlodipine, chlorthalidone) were also prescribed but because of few numbers of such patients, analysis of possible effect of these additional drugs on cognition was not possible. There are certain studies which show that antihypertensive therapy and particularly calcium channel blockers improve the cognitive functions in patients of hypertension.[19]

Some of the limitations of this study are small sample size, not including patients having age more than 60 years and chances of formation of the heterogeneous group because of inclusion of both young and older patients and small duration of the study. Study with large sample size for longer duration having more aged patients should be done to explore this subject further.
